# An Account of Some of the Most Important Diseases Peculiar to Women

**Published:** 1849-01

**Authors:** 


### ARTICLE IX.

*An Account of some of the most important Diseases peculiar to Women.* By Robert Gooch, M. D. With illustrations, second edition, pp. 322. Philadelphia, Ed. Barrington & Geo. D. Haswell, 1848. (From the publishers and for sale by Jos. Keene & Bro., Chicago.)

The style of Dr. Gooch is very entertaining, and frequently amusing; which, added to his strong practical judgment, and clear views, render his small volumes very attractive.

Although embracing a limited range of subjects, the work before us is good on those it treats of, and will make a valuable addition to the practitioner's library. The style of printing we can most cordially recommend on account of its clearness, and the size of the type. Many books are published in such small type, that to read them attentively, and, as physicians generally must, by candle light, most seriously injures the eyes. E.

